# Regulation of FGF2-induced proliferation by inhibitory GPCR in normal pituitary cells

**DOI:** 10.3389/fendo.2023.1183151

**Published:** 2023-07-27

**Authors:** Liliana del V. Sosa, Florencia Picech, Pablo Perez, Silvina Gutierrez, Rodrigo Bainy Leal, Ana De Paul, Alicia Torres, Juan Pablo Petiti

**Affiliations:** ^1^ Universidad Nacional de Córdoba, Facultad de Ciencias Médicas, Centro de Microscopía Electrónica, Córdoba, Argentina; ^2^ Consejo Nacional de Investigaciones Científicas Técnicas (CONICET), Instituto de Investigaciones en Ciencias de la Salud (INICSA), Córdoba, Argentina; ^3^ Universidade Federal de Santa Catarina, Florianópolis, Departamento de Bioquímica e Programa de Pós-graduação em Bioquímica, Centro de Ciências Biológicas, Santa Catarina, Brazil

**Keywords:** pituitary, FGF2, inhibitory GPCR, SST analog, S-phase

## Abstract

**Introduction:**

Intracellular communication is essential for the maintenance of the anterior pituitary gland plasticity. The aim of this study was to evaluate whether GPCR-Gαi modulates basic fibroblast growth factor (FGF2)-induced proliferative activity in normal pituitary cell populations.

**Methods:**

Anterior pituitary primary cell cultures from Wistar female rats were treated with FGF2 (10ng/mL) or somatostatin analog (SSTa, 100nM) alone or co-incubated with or without the inhibitors of GPCR-Gαi, pertussis toxin (PTX, 500nM), MEK inhibitor (U0126, 100µM) or PI3K inhibitor (LY 294002, 10 μM).

**Results:**

FGF2 increased and SSTa decreased the lactotroph and somatotroph BrdU uptak2e (p<0.05) whereas the FGF2-induced S-phase entry was prevented by SSTa co-incubation in both cell types, with these effects being reverted by PTX, U0126 or LY294002 pre-incubation. The inhibition of lactotroph and somatotroph mitosis was associated with a downregulation of c-Jun expression, a decrease of phosphorylated (p) ERK and pAKT. Furthermore, SSTa was observed to inhibit the S-phase entry induced by FGF2, resulting in a further increase in the number of cells in the G1 phase and a concomitant reduction in the number of cells in the S phases (p< 0.05), effects related to a decrease of cyclin D1 expression and an increase in the expression of the cell cycle inhibitors p27 and p21.

**Discussion:**

In summary, the GPCR-Gαi activated by SSTa blocked the pro-proliferative effect of FGF2 in normal pituitary cells *via* a MEK-dependent mechanism, which acts as a mediator of both anti and pro-mitogenic signals, that may regulate the principal effectors of the G1 to S-phase transition.

## Introduction

The anterior pituitary gland is a critical intermediary between the hypothalamus and the entire endocrine system. This gland is regulated by hypothalamic factors and peripheral hormones, as well as by locally produced auto- or paracrine factors, which are the mechanisms controlling hormone secretion as well as proliferation and apoptosis (1–3). Among the myriad of growth factors that regulate the pituitary functionality, the basic fibroblastic growth factor (FGF2) is the most abundant, with the folliculo-stellate cells a non-endocrine pituitary cell type, being its main source (4). FGF2 biological effects are principally mediated by dimerization of the transmembrane tyrosine kinase receptors (FGFR) subtype 1 (5) which they expressed in adenohypophysis cells (6) and we reported the presence of the FGFR1 isoform in the lactotroph cell membrane (7). In neonatal rats, FGF2 plays an important role in the differentiation of lactotroph cells, while in adult rats it participates in the induction of the prolactin gene, secretion of prolactin (PRL) and growth hormone (GH), as well as in lactotroph proliferation (8). In a previous study, we provided evidence that estradiol (E2) and FGF2 exert a cooperative effect on lactotroph proliferation, mediated by mitogen-activated protein kinase MAPK/ERK1/2 as a common signaling pathway (7). Nevertheless, to date the molecular mechanism that modulates the FGF2/FGFR1 effect on anterior pituitary cells proliferation has not yet been fully clarified.

In this sense, previous studies have reported that inhibitory G protein-coupled receptors (GPCR-Gαi) may regulate the intracellular signaling triggered by growth factor receptors transactivation (9–11). Also, a direct association between epidermal growth factor receptor (EGFR) and GPCR- Gαi was shown to down-regulate cell proliferation (12) with FGFR/GPCR-Gαi association reversing the FGF2-induced proliferation in the cell line CHO-K1 (13, 14). In spite of these data, to date the contribution of FGF2-GPCR association on pituitary cell growth control under physiological conditions has not been clarified. Nevertheless, it is unclear whether the SST inhibitory effect is mediated by inhibition of specific hypothalamic releasing factors, or it is a direct effect on pituitary cells.

Focusing on the anterior pituitary physiology, GPCR-Gαi has been shown to be involved in the inhibition of cellular responses induced by endogenous ligands, such as somatostatin (SST) (15, 16). SST has a predominantly inhibitory role as a regulator of pituitary function, mostly by inhibiting hormone secretion and also regulating cell growth. These effects are mediated by the high affinity plasma membrane receptors SSTR1-5, with SSTR5 and SSTR2 being the most abundant in normal pituitary cells (17), which when coupled to pertussis toxin sensitive protein (Gi) induce the inhibition of adenylate cyclase (18). SST analogs (SSTa) have been developed, such as octreotide, which activates the SSTR2 and SSTR5 receptors, but has more affinity to SSTR2 than SSTR5 (19, 20). Cell growth inhibition triggered by SST has been described in pituitary cells *in vivo*, with their administration directly into rat brain ventricles being able to inhibit somatotroph, lactotroph and gonadotroph cell proliferation (21–23). In addition, it was reported that SST inhibited the mitotic activity increase of the pituitary gland with a lack of negative feedback (24). In relation to this, it has been suggested that an increase in the endogenous SST tone may play a role in slowing the proliferative process in hyperplastic pituitaries (25). On the other hand, in a hyperplastic context, it has been suggested that endogenous SST does play a role in suppressing the expansion of the somatotroph population and slowing adenoma formation in response to ectopic GHRH excess. However, this effect has been shown to occur independently of cell proliferation, supporting mechanisms including senescence or apoptosis rather than the inhibition of mitogenesis (26). Bearing this in mind, the SST effects on mitosis in the pituitary gland may contribute to fine adjustment of the size of the pituitary through intracellular mechanisms, although this still need to be clarified along with intracellular communications are essential for modulating pituitary cell proliferation. Thus, this study focuses on evaluating whether GPCR-Gαi modulates the FGF2-induced proliferative activity in normal pituitary cell populations.

## Materials and methods

### Reagents

The basic fibroblast growth factor FGF2 was reconstituted in sterile water to a concentration of 25 ug/mL stock, and the somatostatin analog (SSTa), octreotide was resuspended in sterile water in 1 mM stock, with both solutions being stored at -20°C, thawed once, and the used immediately. Pertussis toxin (PTX), an inhibitor of GαiPCR was resuspended in sterile water in 0.05M stock solution and stored at 4°C. These reagents were purchased from Sigma-Aldrich, St. Louis, MO, United States. The inhibitor of mitogen-activated kinase effector kinase (UO126, Calbiochem, San Diego, USA) was reconstituted in sterile DMSO to a concentration of 10mM.

### Animals

A pool of three-month-old female Wistar rats (n=12 per cell culture) were bred and housed at the Animal Research Facility of National University of Cordoba and assigned to each pituitary cell culture, taken at random estrous cycle stages. The animals were kept in accordance with the Guide for the Care and Use of Laboratory Animals, published by the United States National Institutes of Health (1996), and the experiments were approved by the Institutional Animal Care Committee of the School of Medicine, University National of Cordoba.

### Anterior pituitary cell cultures

The technique used for pituitary cells dissociation and culture have been previously described with modifications (27). Briefly, the rats were euthanized by decapitation within 10 seconds after removal from their cages to avoid any stress or external factors. Then, the anterior pituitaries excised from female rats were placed in minimal essential medium for suspension culture, before being minced, digested with 0.4% trypsin, and dispersed with Pasteur pipettes. The cell yield was 1.5x10^6^ per pituitary, and the cell viability, tested with Trypan Blue exclusion, was always better than 90%. The final suspension was adjusted to 1x106 cells/ml of medium. The dispersed cells were incubated in Dulbecco’s Modified Eagle’s Medium (DMEM-Gibco, NY, US) supplemented with 10% (v/v) fetal bovine serum (FBS) in an oven with a humidified atmosphere of 5% CO2 and 95% air at 37°C.

The anterior pituitary suspension was seeded on glass coverslips (13mm) in 24-well culture plates (1.5x10^5^ cells/mL per well) for BrdU uptake and TUNEL assay or on 6-well culture plates (1x10^6^ cells/mL per well) for to analyse the protein expression by western blotting and cell cycle analysis by flow cytometry.

### Cell treatments and use of inhibitors

The cells were cultured for 3 days in DMEN/10% FBS, in order to in order to provide adequate the cell’s microenvironment. Then incubated with DMEM without serum, supplemented with hydrocortisone (100µg/l), 3,3`-triiodothyronine (400ng/l), transferrin (10mg/l) and sodium selenite (5µg/l) for 24h before treatments. Thereafter, cells were stimulated with FGF2 (10ng/ml) and SSTa (100 nM), either alone or in combination for BrdU uptake, TUNEL and cell cycle by 24 h or for MAPK proteins expression 30 min. In other experiments, cells were pre-incubated with the GαiPCR inhibitor-Pertussis toxin (PTX, 500nM), MEK inhibitor UO126 (100μM) or PI3K inhibitor (10 μM, LY294002) for 30 min for a further 24 h in the same medium. At the end of each experimental condition, the anterior pituitary cells were processed using different techniques.

### Immunocytochemical detection of bromodeoxyuridine uptake

Cells at the DNA synthesizing stage were identified by immunocytochemical detection of 5-bromo-2’-deoxyuridine (BrdU) uptake in cell monolayers grown on coverslips. Pituitary cell cultures were stimulated for 24h with the compounds mentioned above, and BrdU (100nM, Sigma-Aldrich) was added during the stimulation. Then, normal pituitary cells attached to the coverslips were fixed in 4% formaldehyde in PBS for 2h at room temperature (RT) and BrdU detection was performed. Briefly, after fixation retrieval of the antigen was achieved by treating the slides in 10 mM citrate buffer (pH 6.0) in a hot water bath (95°C) for 5 min in microwave. For digestion of cellular DNA, cells were incubated with freshly prepared DNase I (1 U/uL) for 30 min at 37°C in a wet chamber. After incubation with 5% BSA in PBS-Triton for 30 min, sections were incubated with anti BrdU antibody (1/200) overnight at 4°C. Sections were washed and incubated with an anti-mouse Alexa 488 or 594 secondary antibody (1/3000 Invitrogen, Carlsbad, CA US). This was followed by DAPI incubation (Sigma-Aldrich, St. Louis, MO US) for 10 minutes. Images were obtained using a FluoView FV 1000 microscope, these were analyzed using ImageJ software 1.51 (Wayner Rasband National Institute of Health, USA). A total of 1000 cells were quantified on each glass slide using a systematic process to establish the percentage of positive BrdU with respect to all pituitary cells.

In addition, PRL or GH immunocytochemistry for lactotroph or somatotroph identification was performed on the same coverslip. Cells were incubated with rabbit anti-PRL (1/3000, NIDDK-rPRL-IC-5) or anti- GH (1/1000, NIDDK-rGH) in a wet chamber for 1h at 37°C, washed in PBS and incubated with anti-rabbit Alexa Fluor 594 antibody (1:3000, Invitrogen) for 1h at 37°C. Controls were also performed, by applying the same protocols, but omitting BrdU, PRL or GH primary antibodies. Images were obtained using a FluoView FV 1000 microscope, these were analyzed using ImageJ software 1.51 (Wayner Rasband National Institute of Health, USA).

A total of 1000 PRL or GH immunoreactive cells were quantified on each glass slide by fluorescence light microscopy using a systematic process, standardized in our laboratory, to establish the proportion of double-positive BrdU-PRL or BrdU-GH cells in the total PRL- or GH-positive cells.

### TUNEL assay

Cell death was assessed by applying the TUNEL technique using the *In Situ* Cell Death Detection Kit (Roche) for nick-end labelling detection following the manufacturer’s instructions, as previously described (28). For the negative controls, samples were incubated with label solution omitting the enzyme solution, while for the positive control, cells were treated with DNase I. After mounting with glycerol, the cells attached to coverslips were observed and photographed using a Zeiss Axiostar plus microscope at 400X. For quantification of the cells, 1000 cells/coverslip were counted to obtain the percentage of apoptotic cells per treatment.

### Preparation of cell lysates for western blotting

Pituitary cells were lysed in cold lysis buffer (RIPA buffer with a cocktail of proteases and phosphatases inhibitors). Proteins from the total homogenate (40µg) were separated using 12% polyacrylamide gel and transferred to a nitrocellulose membrane. Unspecific binding was blocked with 5% non-fat dried milk and 0.1% Tween20 at RT and incubated overnight with the primary antibodies: 1/1000 anti-SSTR2 (sc-365502, Santa Cruz Biotechnology), 1/4000 anti-SSTR5 (#PA3-209, Thermo Fisher Scientific), 1/1000 anti-diphosphorylated ERK1/2 (p-ERK1/2; Cell Signaling) 1/500 anti-total ERK1 (T-ERK1/2; Santa Cruz Biotechnology, Inc),1/1000 anti-phosphorylated JNK (p-JNK), 1/1000 anti-phosphorylated p38 MAPK (p-P38; Millipore), 1/10000 anti-total-p38 MAPK (T-P38), 1/5000 anti-total JNK (T-JNK), 1/1000 anti-c-Jun (Cell Signaling), 1/500 anti-phosphorylated AKT (p-AKT; Cell Signaling), 1/2000 anti-total Akt (T-AKT), 1/1000 anti-phosphorylated PS6 (p-PS6; Cell Signaling), 1/300 anti-CyclinD1 (CD1; Cell Signaling), 1/500 anti-CyclinE1 (CE1; Santa Cruz Biotechnology, Inc), 1/500 anti-CDK4 (abcam), 1/1000 anti-p21(Cell Signaling), 1/500 anti-p27 or 1/4000 anti-β-Actin (Sigma-Aldrich). The blots were incubated with peroxidase-conjugated anti-rabbit (1/5000) or anti-mouse (1/2500 Jackson Immunoresearch Labs Inc, PA, USA) secondary antibodies and then revealed with ECL detection reagents (Inmun-Star HRP-Substrate Kits, Bio-Rad, CA, USA). Finally, the emitted light was captured on hyperfilm (Amersham Pharmacia) and signals were quantified with ImageJ software.

### Double immunofluorescence

Pituitary cell cultures were stimulated for 24h with the compounds mentioned above, and normal pituitary cells attached to the coverslips were fixed in 4% formaldehyde in PBS for 2h at room temperature (RT). For immunolabelling, after washing and blocking, the samples were incubated with anti-pERK1/2 (1/500) and anti PRL (1/3000 NIDDK-rPRL-IC-5) ON at 4°C, and rinsed and incubated with anti-rabbit Alexa-Fluor 594 (1/3000 Invitrogen, Carlsbad, CA US) or anti-rabbit Alexa-Fluor 488 (1/2500 Invitrogen, Carlsbad, CA US) for 1h at 37°C. This was followed by DAPI incubation (Sigma-Aldrich, St. Louis, MO US) for 10 minutes. Images were obtained using a FluoView FV 1000 microscope, these were analyzed using ImageJ software 1.51 (Wayner Rasband National Institute of Health, USA).

### Cell cycle analysis by flow cytometry

Pituitary cells were harvested, washed in PBS, fixed with ethanol 70%, and incubated with RNAse (10 ug/ul; Ribonuclease A, Sigma R5503; St Louis, USA), for 15min at 37°C, after which, DNA was stained with propidium iodide (50 ug/ml; Sigma P4170; St Louis, USA) to analyze the cellular DNA content. Cell cycle analysis was performed on a Coulter flow cytometer (BD FACS Canto II), in order to determine the percentage of cells in the G1, S and G2/M phases.

### Data analysis

The results were expressed as mean ± S.E.M measured from three independent cell cultures. Statistical analysis was performed using GraphPad Prism version 8.0.2. One-way ANOVA followed by Tukey’s test was used with a significance level of p<0.05 for BrdU uptake and western blot assays; or two-way ANOVA followed by Bonferroni’s test with a significance level of p<0.05 for BrdU uptake assays with or without different inhibitors.

## Results

### SSTa inhibits the FGF2 S-phase entry in normal lactotroph and somatotroph cells

**Figure 1:**
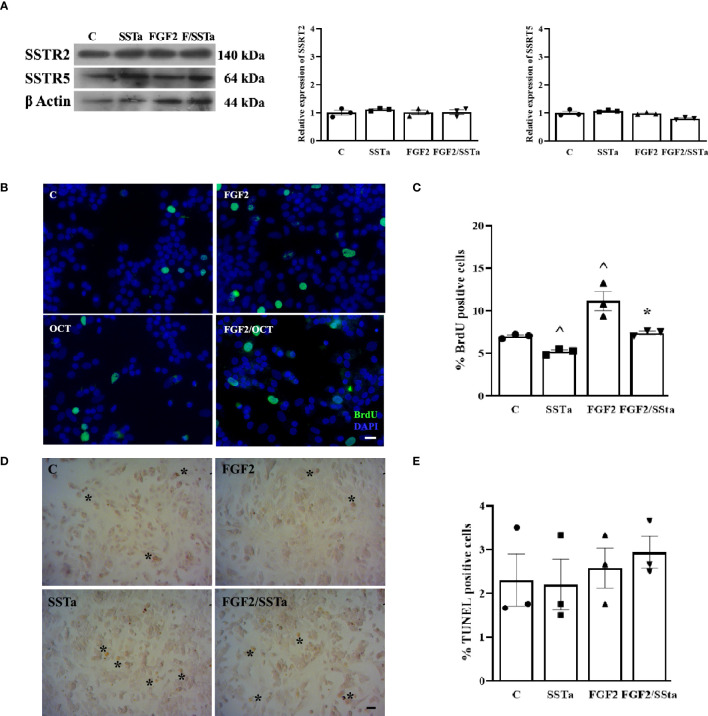
The FGF2- induced S-phase entry was inhibited by SSTa in the anterior pituitary cell culture. **(A)** Primary pituitary cells were incubated with FGF2 (10ng/ml) and SSTa (octreotide, 100 nM), either alone or in combination for 24h in fresh serum-free medium. Representative immunoblots and quantification of SSTR2 and SSTR5 protein expression in total extract of anterior pituitary cell culture (n=3). **(B, C)** Primary pituitary cells were incubated with FGF2 (10ng/ml) and SSTa (octreotide, 100 nM), either alone or in combination for 24 h, and BrdU was added during the stimulation Representative micrographs and quantification of BrdU uptake (green nucleus) represent the proportion of positive BrdU-labeled cells among the total cells. The data are presented as means ± SE of 3 wells from three independent experiments (n=9), and the statistical analysis was conducted using one-way ANOVA-Tukey: ^p <0.05 vs control **(C)** and *p<0.05 vs FGF2. Scale bar: 20 μm. **(D, E)**. Cell death was detected using the TUNEL assay and quantified in the anterior pituitary cell culture treated with FGF2 (10 ng/mL) and SSTa (octreotide, 100 nM) alone or in combination for 24 hours (n=3). Scale bar: 20 μm.

First, we analyzed by western blot the SSTR2 and SSTR5 protein expression levels in primary pituitary cell cultures exposed to SSTa or FGF2 for 24h. As shown in **Figure 1A**, neither FGF2 or SSTa alone nor in co-incubation modified the expression of the GPCR-Gαi receptors SSTR2 or SSTR5. Bearing in mind that we previously reported FGF2 proliferative effects on pituitary cells *in vitro* (7), we now analyzed whether the SSTa treatment could prevent this type of S-phase entry, with the SSTa treatment being observed to significantly decrease the BrdU positive cell number, while FGF2 exposure increased cell BrdU uptake compared to control, an effect that was reverted in the presence of SSTa treatment (p<0.05) and reached similar values to those of control (**Figures 1B, C**).

After detecting a FGF2/SSTa-reduced pituitary BrdU uptake, we next evaluated the impact of the treatments on cell death by determining the percentage of apoptosis using the TUNEL assay. As shown in **Figures 1D, E**, no changes were observed after any treatments over 24h.

**Figure 2:**
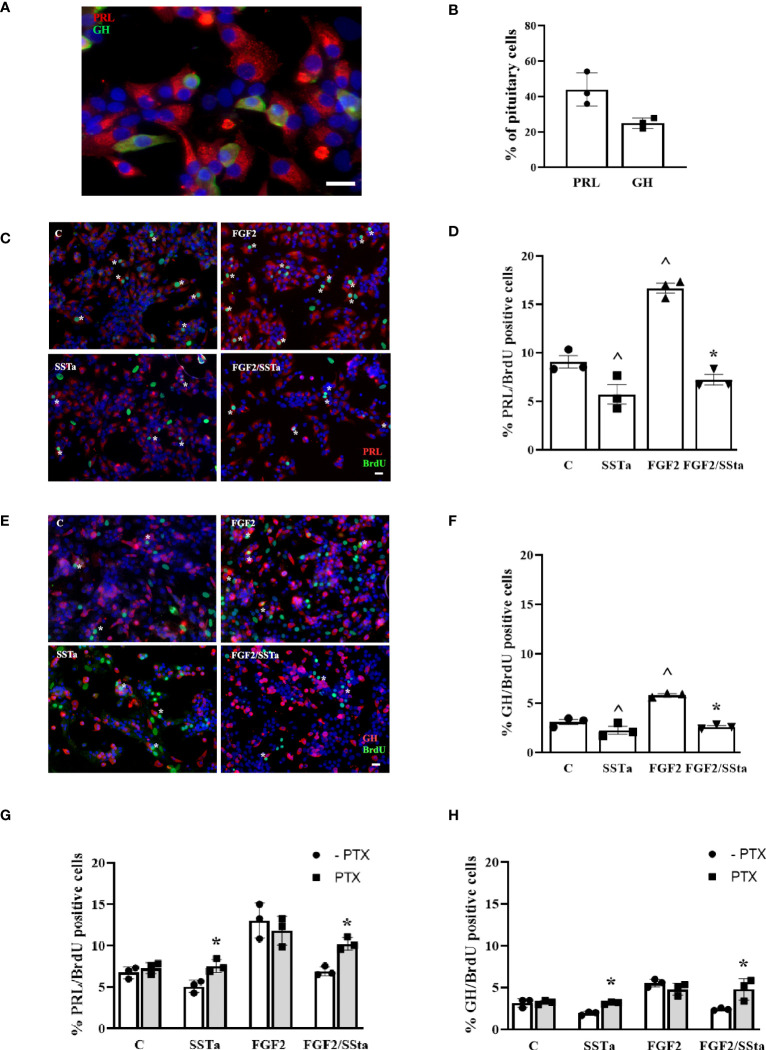
The FGF2 induced S-phase entry was inhibited by SSTa in lactotrophs and somatotroph cells. **(A)** Double-immunocytochemistry of PRL (red) and GH (green) was performed in an anterior pituitary cell culture. **(B)** The percentages of PRL and GH cells in the anterior pituitary cell culture were determined using flow cytometry (n=3). Primary pituitary cell were incubated with FGF2 (10ng/ml) and SSTa (octreotide, 100 nM), either alone or in combination for 24 h, and the BrdU was added during the stimulation. **(C, D)** Representative micrographs and quantification of BrdU uptake in PRL cells were performed. The data represent the proportion of double-positive cells for BrdU (green) and PRL (red) among the total PRL-positive cells. The data are presented as means ± SE of 3 wells from three independent experiments (n=9), and statistical analysis was conducted using one-way ANOVA-Tukey: ^p <0.05 vs control **(C)** and *p<0.05 vs FGF2. Scale bar: 20 μm. **(E, F)** Representative micrographs and quantification of BrdU uptake in GH cells were also conducted. The data represent the proportion of double-positive cells for BrdU (green) and GH (red) among the total GH-positive cells. The data are presented as means ± SE of 3 wells from three independent experiments (n=9), and statistical analysis was conducted using one-way ANOVA-Tukey: ^p <0.05 vs control **(C)** and *p<0.05 vs FGF2. Scale bar: 20 μm. Primary pituitary cell cultures were pre-treated with pertussis toxin (PTX, 500nM) for 30min and then the cells were stimulated with FGF2 (10ng/mL) and SSTa (octreotide, 100nM) alone or co-incubated for 24h, and the BrdU was added during the stimulation. **(G)** Quantification of BrdU uptake in PRL cells, representing the proportion of double-positive cells for BrdU and PRL among the total PRL-positive cells. **(H)** Quantification of BrdU uptake in GH cells, representing the proportion of double-positive cells for BrdU and GH among the total GH-positive cells. The data are presented as means ± SE of 3 wells from three independent experiments (n=9), and statistical analysis was conducted using two-way ANOVA-Bonferroni. *p<0.05 SSTa vs SSTa with PTX or FGF2/SSTa vs FGF2/SSTa with PTX.

Considering that SSTa reduced FGF2-induced S-phase entry and, taking into account that the lactotroph and somatotroph cells are the two principal pituitary cell populations, we analyzed the BrdU uptake response to different treatments in these two main cell populations. Under the present experimental conditions, lactotrophs and somatotrophs represented 43.90± 9.31% and 24.88± 2.98% respectively, of the total pituitary cells, as quantified by immunofluorescence and flow cytometry (**Figures 2A, B**), with the percentage of lactotroph or somatotroph-BrdU positive cells being the 9.05 ± 0.64% and .08 ± 0.27%, respectively in control conditions. As expected, FGF2 significantly increased PRL and GH cell BrdU uptake by 16.66 ± 0.51% and 5.83 ± 0.12% respectively, an effect that was reduced when both pituitary cell types were exposed to SSTa simultaneously (7.25 ± 0.54% PRL and 2.59 ± 0.13% GH) (**Figures 2C–F**).

To try to verify the involvement of the GPCR-Gai pathway in the SSTa inhibitory effect on FGF2-induced S-phase entry, pituitary cell cultures were pre-incubated with PTX (an inhibitor of GPCR-Gαi) for 30min prior to the addition of SSTa or FGF2, either alone or in co-incubation, for an additionally 24h. Then, the BrdU uptake in lactotrophs and somatotrophs was analyzed. This inhibitor was found to be able to revert the SSTa effect on BrdU uptake, both alone or with FGF2 co-incubation, and reached similar values to those of control. This increased cell BrdU uptake in lactotrophs or somatotroph cells induced by FGF2, was not modified by PTX pre-incubation (**Figures 2G, H**).

### Inhibition of the FGF2 S-phase entry response by SSTa was associated with a modulation of the MEK and PI3K pathways

**Figure 3:**
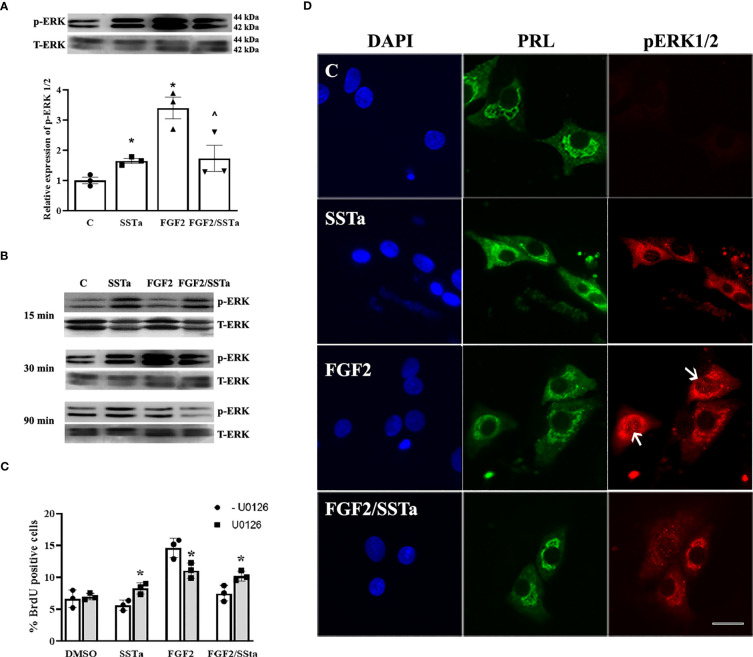
Regulation of ERK 1/2 by FGF2 and SSTa alone or co-incubated. **(A)** Representative immunoblots and densitometric analysis of pERK1/2-T-ERK1/2, after 30 min of treatment with FGF2 (10ng/mL) and SSTa (octreotide, 100nM) alone or co-incubated in anterior pituitary cell culture. The data are presented as mean ± SE of three independent experiments (n=3), and the graph represents the pERK1/2-T-ERK1/2 ratio. Statistical analysis was conducted using one-way ANOVA-Tukey: *p<0.05 vs control and ^p<0.05 vs FGF2. **(B)** Immunoblots of pERK1/2-T-ERK1/2 after 15, 30, and 90 minutes of treatment with FGF2 (10 ng/mL) and SSTa (octreotide, 100 nM), either alone or in combination, in anterior pituitary cell culture. **(C)** Primary pituitary cell cultures were pre-treated with the MEK inhibitor UO126 (100 μM) for 30 minutes. The cells were then stimulated with FGF2 (10 ng/mL) and SSTa (octreotide, 100 nM), either alone or in combination, for 24 hours, with BrdU added during the stimulation. Quantification of BrdU uptake represents the proportion of positive BrdU-labeled cells among the total cells. The data are presented as means ± SE of three wells from three independent experiments (n=9), and statistical analysis was conducted using two-way ANOVA-Bonferroni: *p<0.05 vs FGF2, SSTa, or FGF2/SSTa without UO126. **(D)** Representative microphotographs of double-immunocytochemistry of pERK (red) and PRL (green) in anterior pituitary cell culture, incubated with FGF2 (10 ng/mL) and SSTa (octreotide, 100 nM), either alone or in combination, for 5 minutes (n=3). The arrows indicate pERK1/2 in the nucleus of PRL-positive cells. Scale bar: 20 μm.

To determine whether MAP kinases such as c-jun N-terminal kinase (JNK), p38, and ERK1/2 pathways were associated with the SSTa effects on FGF2-induced S-phase entry in an anterior pituitary cell culture we analyzed the expression of each protein by western blot. As shown in **Figure 3A**, a significant increase in pERK1/2 protein expression levels was detected after SSTa and FGF2 treatment alone compared to control. In addition, ERK1/2 phosphorylation decreased when the pituitary cells were treated with FGF2/SSTa for 30 min (p<0.05 vs FGF2 treatment).

In order to understand the ERK1/2 activation in pituitary cells under pro- and anti-proliferative signals, the pERK1/2 expression in primary cell cultures treated with SSTa or FGF2 alone or in-coincubation for 15, 30 and 90 min was determined. **Figure 3B** shows that pERK1/2 increased in response to SSTa, but not in FGF2-stimulated cells at 15 min. However, at 30 minutes of incubation, both treatments increased pERK1/2, with FGF2 showing a more pronounced effect, and returning to basal levels after 90 min. In addition, the pERK1/2 expression in PRL positive cells which constitute the main population in the pituitary cell culture, was analyzed using double immunofluorescence. As shown in **Figure 3D**, there was a low and sparse labeling of pERK1/2 in the cytoplasm of PRL cells under control conditions. However, after incubation with FGF2 for 5 min, the signal of MAP kinases became more pronounced and localized in the nucleus, an effect that was reversed in the presence of SSTa.

To try to verify the role of MAPK-ERK1/2 on the SSTa anti-proliferative effect, pituitary cell cultures were pre-incubated with UO126 (inhibitor of MEK) for 30min prior to the addition of SSTa or FGF2 applied alone or in co-incubation, for a further 24h. The MEK inhibitor was able to revert partially the SSTa inhibitory effect on FGF2-induced S-phase entry (p<0.05) (**Figure 3C**).

**Figure 4:**
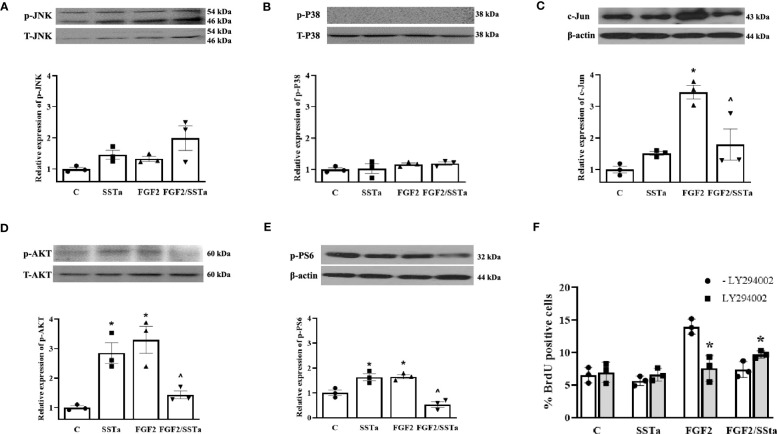
Regulation of MAPK and PI3K pathway by FGF2 and SSTa alone or co-incubated. Representative immunoblots and densitometric analysis of **(A)** pJNK-T-JNK, **(B)** p-P38-TP38 **(D)** pAKT-T-AKT and **(E)** pPS6-b-actin after 30 min of treatment with FGF2 (10ng/mL) and SSTa (octreotide, 100nM) alone or co-incubated in anterior pituitary cell culture. The data shown represent the mean ± SE of three independent experiments (n=3), and the graph represents the ratio of pJNK-T-JNK, p-P38-TP38, pAKT-T-AKT, and pPS6-b-actin. Statistical analysis was performed using one-way ANOVA-Tukey: *p<0.05 vs control and ^p<0.05 vs FGF2. **(C)** Representative immunoblots and densitometric analysis of c-Jun/b-actin after 24h-treatment with FGF2 (10ng/mL), SSTa (octreotide, 100nM) or the combination. Results are expressed as mean ± SEM, and determinations were repeated three times. The graph represents the c-Jun/b-actin ratio, and statistical analysis was conducted using one-way ANOVA-Tukey: *p<0.05 vs control and ^p<0.05 vs FGF2. **(F)** Primary pituitary cell cultures were pre-treated with PI3K inhibitor LY294002 (100μM) for 30min and then the cells were stimulated with FGF2 (10ng/mL) and SSTa (octreotide, 100nM) alone or co-incubated for 24h, and the BrdU was added during the stimulation. The data are presented as means ± SE of three wells from three independent experiments (n=9), and statistical analysis was performed using two-way ANOVA-Bonferroni: *p<0.05 vs FGF2 or FGF2/SSTa without LY294002.

On the other hand, we analyzed JNK and p38 phosphorylated proteins expression without any significant differences being observed (**Figures 4A, B**). In addition, to gain a better insight into the molecular mechanism by which SSTa inhibited the pituitary cell growth induced by FGF2, we examined the expression of the transcription factor c-Jun, known downstream effector of the pERK1/2 pathway (29). **Figure 4C** shows that the FGF2/SSTa combined treatment inhibited c-Jun protein expression, respect to FGF2 treatment alone.

The activated FGF receptors have been coupled to intracellular signaling pathways, including the RAS-MAPK, PI3K-AKT, PLCγ, and STAT pathways, which participate in the cell proliferation in different cell types (5). In our experimental conditions, the MEK inhibitor partially abolishes the stimulatory effect of FGF2 on cell proliferation, suggesting that additional pathways besides the ERK pathway are involved in promoting cell proliferation. In this sense, pituitary primary cell cultures were pre-incubated with the PI3K inhibitor (LY294002; LY, 10 μM) for 30 min before the addition of SSTa or FGF2, applied alone or in co-incubation for an additional 24h and the BrdU uptake was analysed. As shown in **Figure 4F**, the S-phase entry induced by FGF2 was reverted, while the inhibitory effect of SSTa on FGF2-induced proliferation was partially reverted with LY pre-incubation. In addition, the PI3K/AKT pathway and downstream target phospho-S6 (p-PS6) were analyzed after FGF2 or SSTa alone or in co-incubation with FGF2 for 30 minutes. **Figures 4D, E** shows that FGF2 and SSTa increased the phosphorylation of AKT and PS6, an effect that was reversed with the FGF2/SSTa co-incubation. These results suggest that both the MEK/ERK1/2 and PI3K/AKT pathways may regulate pituitary cell proliferation by mediating pro- and anti-mitogenic signals.

### The FGF2 proliferative effect was regulated by SSTa-induced cell cycle arrest in anterior pituitary cells

**Figure 5:**
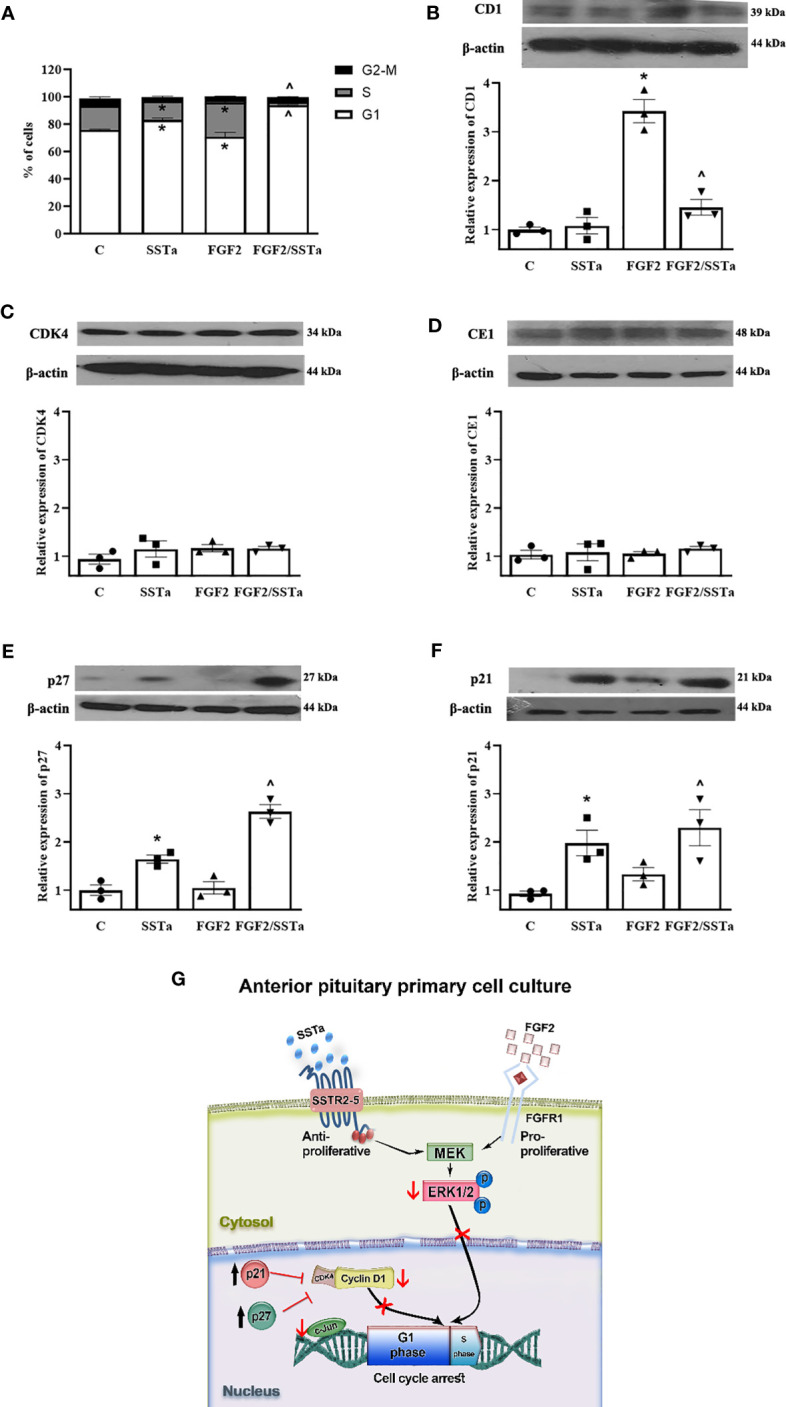
The FGF2 and SSTa combination treatment induced cell cycle arrest in anterior pituitary cell culture. **(A)** Primary pituitary cells were incubated with FGF2 (10 ng/mL) and SSTa (octreotide, 100 nM), either alone or in combination, for 24 hours. The cell cycle phases (G1, S, and G2-M) in anterior pituitary cells were determined by flow cytometry, and the percentage of cells in each phase of the cell cycle after different treatments is shown. Data are represented as mean ± SD of three independent experiments and were evaluated by one-way ANOVA-Tukey: *p<0.05 vs control **(C)**, ^p<0.05 vs FGF2. Representative immunoblots and densitometric analysis of **(B)** cyclin D1, **(C)** CDK4, **(D)** cyclin E1, **(E)** p27, and **(F)** p21 protein expression after 24h of treatment with FGF2 (10ng/mL) and SSTa (octreotide, 100nM) alone or co-incubated in the anterior pituitary cell culture. The data shown represent the mean ± SE of three independent experiments (n=3), and the graphs represent the cyclin D1/b-actin, CDK4/b-actin, cyclinE1/b-actin, p21/b-actin, and p27/b-actin ratios. Statistical analysis was performed using one-way ANOVA-Tukey: *p<0.05 vs control **(C)**, ^p<0.05 vs FGF2. **(G)** Model of p-ERK1/2 regulating anterior pituitary primary cell proliferation by FGF2 and SSTa. FGF2 induces ERK phosphorylation and nuclear translocation to induce cyclin D1 expression and S-phase entry, an effect that is reversed by SSTa.

Having demonstrated that SSTa inhibited the BrdU uptake triggered by FGF2, next we analyzed the effects on cell cycle progression. As expected, FGF2 induced an increase in the number of cells in the S phase, concomitantly accompanied by a decrease of cells in the G1 phase (p < 0.05). SSTa increased the cell number in the G1 stage (p < 0.05), with a corresponding reduction of pituitary cells in the S phase (p <0.05). Interestingly, the SSTa/FGF2 treatment revealed a significant increase in the pituitary cell number in the G1 phase, with a concomitant reduction of cells in the S phase (p< 0.05) (**Figure 5A**). Next, we analyzed the expression of the key proteins involved in the early phases of cell cycle progression (G1 to S-phase transition). As shown in **Figure 5B**, FGF2/SSTa co-incubation induced a marked decrease in the cyclin D1 protein levels (p<0.05 vs FGF2), while cyclinE1 and CDK4 remained unchanged (**Figures 5C, D**). In addition, the expression of the CDK inhibitors was modulated by FGF2/SSTa co-incubation, with an increase of p27 and p21 levels being observed (**Figures 5E, F**).

## Discussion

A main characteristic of the anterior pituitary gland is its plasticity, which allows it to adjust to different physiological conditions related to endocrine demands (2, 3, 30). This property is associated with the adjustment and maintenance of the anterior pituitary cell number mediated by specific local growth factors, which may be regulated by stimulatory or inhibitory extra-pituitary factors. In this context, in the present work, we addressed the question whether the activation of GPCR-Gαi induced by somatostatin analog (SSTa) could regulate the FGF2 proliferative activity in normal pituitary cell populations. Our findings showed that FGF2 increased whereas SSTa decreased pituitary cell BrdU uptake, specifically in lactotroph and somatotroph cell populations. Interestingly, the combined treatment of both factors (FGF2/SSTa) resulted in a decrease in lactotroph and somatotroph cell populations. This response was associated with the arrest of the cell cycle in the G1 stage, a reduction of ERK1/2 and AKT phosphorylation proteins compared to FGF2, thereby, demonstrating the involvement of GPCR- Gαi in the modulation of the S-phase entry induced by FGF2 in lactotroph and somatotroph cells.

The main somatostatin (SST) effect on the pituitary function is the control of hormone secretion due to acute inhibition of hormone exocytosis, but a secondary effect concerning the regulation of cell proliferation has also been described. It has been suggested that the SST anti-proliferative effect might be mediated through the inhibition of specific hypothalamic releasing factors or by its direct effect on pituitary cells (31). The inhibition of cell growth induced by SST has been demonstrated in normal and tumoral pituitary cells *in vivo*, which blocked somatotroph, lactotroph, gonadotroph and thyrotroph cell proliferation when SST was administrated directly into rat brain ventricles (21–23). In the present study, our results showed that SSTa prevented FGF2 S-phase entry in normal primary cell culture in lactotroph and somatotroph cells. It has also been reported that the SST agonist octreotide prevented pituitary cell proliferation, induced by estradiol, suggesting that activation of the SSTRs modulated the pro-proliferative signals (32, 33).

There is evidence that SSTR not only has cytostatic but also cytotoxic action (34). However, we did not observe apoptosis with either SSTa or after the combined treatment of FGF2/SSTa under our experimental conditions. Here, the SSTa inhibition of BrdU uptake induced by FGF2 was associated with an increase in the number of cells in the G1 phase and a concomitant reduction in the number of cells in the S phase. In agreement with this, a cytostatic effect on the GH3 pituitary tumor cells was observed, which exhibited a partial inhibition of the cell cycle progression from the G0/G1 to S phase with no apoptosis occurring after incubation with somatostatin or octreotide in serum-supplemented media (35). In addition, in other cell types, the expression of ligand-activated SSTR2 and SSTR5 was capable of inhibiting the mitogenic signal of serum or growth factors and inducing G1 cell cycle arrest (36, 37). The effects of these important regulators may contribute to metabolic homeostasis and might provide a physiological benefit in pituitary cells (38).

The antiproliferative effect of SST can be mediated by the five SSTR, according to receptor subtypes and target cells (39, 40). In the pituitary gland, SSTR2 and SSTR5 are the most abundantly expressed SSTRs (41) Here, we demonstrated that SSTa inhibited the proliferative effect induced by FGF2 in lactotroph and somatotroph cells which expressed both receptors. This effect could be mediated by a major contribution of SSTR2, since octreotide has a high affinity for SSTR2 and a low affinity for SSTR5. However, due to this, SSTa exhibits an effective overlapping binding for more than one receptor subtype, it is difficult to distinguish between receptor-selective pathways upon SSTa stimulation. Cattaneo et al, have reported that a combined stimulation of SSTR2 and SSTR5 was necessary to obtain a significant inhibitory effect, suggesting a possible heterodimerization between both receptors (42). In addition, it has been described that SSTR2 and SSTR5 synergize functionally to inhibit GHRH-induced GH secretion in human fetal pituitary cultures (43). Although SSTR physical dimerization in pituitary cells has not been reported (31), it is possible to suggest that a functional interaction between receptor signaling pathways could be important, thereby contributing to the complexity of SST action in pituitary cells.

Ligand binding to SSTRs triggers the activation/inhibition of cytoplasmic targets, leading to multiple intracellular signaling pathways, including -pertussis toxin sensitive (Gi dependent) (34), suggesting the involvement of GPCR-Gαi in the inhibition of cellular responses induced by endogenous ligands such as somatostatin. In the present study we observed that the MEK inhibitor partially blocked the inhibitory effect of SSTa on FGF2-induced BrdU uptake, implying a possible role of ERK1/2 phosphorylation mediating the SSTR2 and SSTR5 anti-mitogenic functions. Concerning ERK1/2, we observed that inhibition with U0126 blocked not only the anti-proliferative effect of SSTa, but also the pro-proliferative response of FGF2. The ERK1/2 pathway contribute to cell cycle progression and cell survival (44), with mutations activation in the constituents of the RAS-ERK pathway being common in neoplasia (45). However, a sustained activation may also induce cell cycle arrest or cell death in various cell types (46). These paradoxical functions of ERK1/2 are mainly determined by the magnitude and duration of their catalytic activity, spatio-temporal regulation, and monomeric *vs*. dimeric status, as well as physical interactions with effectors after translocation to different subcellular compartments (47). Here, we suggest that ERK1/2 may play a pivotal role in maintaining pituitary homeostasis through mediating the pro- and anti-proliferative signals triggered by FGF2 and SSTa respectively. In addition, FGF2 induced sustained activation of ERK1/2, while the somatostatin analog generated both transient and sustained ERK1/2 activation. Thus, we hypothesize that the decision to proliferate in pituitary cells exposed to antagonistic stimuli may be regulated by the different pulses of pERK1/2, which in turn modify ERK-interacting proteins. Through quantitative proteomics, 284 ERK-interacting proteins have been identified, and these interactions change differentially after growth factor stimulation in the same biological context (48). These data demonstrate that the decision to proliferate is an integrative process controlled at every step of the signaling cascade by multiple mechanisms, including regulation of ERK activation kinetics, subcellular translocation, and differential substrate phosphorylation (49).

It has been described that about 30% of rat pituitary cells arise from “self-mitosis” of already differentiated cells, or possibly from pituitary stem cells (50). One plausible hypothesis is that the hypothalamus regulates cell turnover in the pituitary, either as a whole or in terms of individual processes (51). In this sense, intracerebroventricular administration of somatostatin in rats has been shown to inhibit lactotroph and somatotroph cell proliferation (21). However, endogenous SST primarily controls GHRH-induced adenoma formation *via* modulation of apoptotic and/or cellular senescence pathways in a hyperplastic context (26). In the present study, FGF2 induced cell cycle progression from the G1 to S phase, with this effect being blocked when the pituitary cells were treated with this growth factor in the presence of the somatostatin analog, in agreement with results obtained by BrdU assays. Our findings suggest that somatostatin may counterbalance the pro-mitogenic response of FGF2 to prevent an excessive proliferative response in pituitary homeostasis.

The regulation of cell cycle progression involves multiple molecular mediators which are necessary to preserve pituitary homeostasis. Different mouse models of the cell cycle-related proteins involved in pituitary biology have been developed over the years (52). Cyclin D1 acts as sensor of multiple mitogenic signals to activate CDK4 and to induce the progression during G1 phase (52), being described that ERK activation induces cyclin D1 expression (53). In this regard, the relationship between ERK activation, cyclin D1 transcription, and S-phase entry has been demonstrated. The inhibition of ERK activity by a pharmacological MEK inhibitor completely prevents the increase in cyclin D1 protein levels and S-phase entry in human cells incubated with FGF2 (54). Interestingly, ERK1/2 activity is not only important for inducing S-phase entry, but it also requires translocation to the nucleus (55). Experiments strongly suggest that ERK1/2 nuclear translocation is a crucial step in signal transduction that leads to gene expression and promotes cell cycle re-entry upon mitogenic stimulation (54). In our study, we observed that FGF2 induced ERK1/2 nuclear translocation, an effect that was reversed in the presence of SSTa. In addition, we showed that the G1 arrest observed after the co-incubation with FGF2 and SSTa was related to a significant decrease of Cyclin D1 expression, without any alterations being detected in CDK4 levels. Our results suggest that SSTa may modulate FGF2-induced S-phase entry by regulating ERK1/2 activation, nuclear translocation, and consequently decreasing Cyclin D1 expression. The activity of the dimeric cyclin-CDK complex can be inhibited by the CDK interacting protein/kinase inhibitory protein (CIP/KIP) family of protein inhibitors, such as p21^Cip1^ and p27^Kip1^ (56). We observed that the G1 arrest induced by SSTa, both alone and in combination with FGF2, was associated with an increase in p21 and p27 protein levels. The relevance of both inhibitors in pituitary homeostasis has been demonstrated in transgenic mice, with both p27^Kip1^ and p21^Cip1^ deficiency accelerating pituitary tumorigenesis (57, 58). Related to this, our results suggest that the stimulation of inhibitory GPCRs counterbalances the cell cycle progression triggered by growth factors modulating the levels of Cyclin D1, p21 and p27 in a physiological pituitary context.

In pituitary cells, one crucial decision in order to maintain homeostasis is whether to re-enter to cell cycle or to reach a non-proliferative state, which depends on the different receptors activated. The MAP kinase signal transduction pathways play an important role in the regulation of the cell cycle in mammalian cells in a complex manner by sharing substrates and cross-cascade interactions (59). However, the same signaling pathway may have opposite functions in the same cell. It has been demonstrated that a low-level activation of Raf/MEK/ERK1/2 leads to the induction of cyclin D1 and to reduced p27^Kip1^ expression, thereby promoting the entry of quiescent cells into the cell cycle. In contrast, higher levels of Raf/MEK/ERK1/2 activity lead to induction of p27^Kip1^ and p21^Cip1^ expression, which inhibits the cyclin D-CDK4 complex, thereby eliciting cell cycle arrest (60). In pituitary cells, we suggest that the high ERK1/2 activation observed after FGF2 treatment induced cell cycle progression by increasing cyclin D1 levels, but when GPCR-Gαi was activated, the level of phosphorylated ERK1/2 decreased, and the cells were arrested in G1 as a consequence of an increase of both p21^Cip1^ and p27^Kip1^ expression.

In summary, in normal pituitary cells, GPCR-Gαi activated by SSTa blocked the S-phase entry effect of FGF2 in normal pituitary cells via a molecular mechanism that involved activation of ERK1/2 as a hub mediator of both, anti and pro-mitogenic signals, which may regulate the main effectors of the G1 to S phase transition (**Figure 5G**).

## Data availability statement

The original contributions presented in the study are included in the article/. Further inquiries can be directed to the corresponding authors.

## Ethics statement

The animal study was reviewed and approved by Institutional Animal Care Committee of the School of Medicine, University National of Cordoba.

## Author contributions

The authors have made the following declarations about their contributions: Conceived and designed the experiments: LS, AT and JP. Performed the experiments: LS, FP, PP. Analyzed the data: LS, FP, PP, SG, RL. Manuscript preparation: LS, SG, RL, AD, AT, and JP. All authors contributed to the article and approved the submitted version.
